# On the link between cell cycle and infection of the Alphaproteobacterium *Brucella abortus*

**DOI:** 10.15698/mic2014.10.171

**Published:** 2014-09-29

**Authors:** Michaël Deghelt, Jean-Jacques Letesson, Xavier De Bolle

**Affiliations:** 1 URBM, UNamur, Belgium.

**Keywords:** Brucella, Caulobacter, bacterial cell cycle, cellular infection, intracellular trafficking

## Abstract

Bacteria of the *Brucella* genus are responsible for brucellosis, a worldwide zoonosis. These bacteria are known to have a peculiar intracellular trafficking, with a first long and non-proliferative endosomal stage and a second proliferation stage, often associated with its localization of the bacteria in the endoplasmic reticulum (ER). However, the status of the bacterial cell cycle during the non-proliferative phase was still unknown. In a recent study [Nat. Communic. 5:4366], we followed the cell cycle of *B. abortus* in culture and inside the host cells. In culture, *B. abortus* initiates the replication of its large chromosome before the small chromosome. The origin and terminator regions of these two chromosomes display distinct localization and dynamics within *B. abortus*. In HeLa cells and RAW264.7 macrophages, the bacteria in G1 (i.e. before the initiation of chromosomes replication) are preferentially found during the endosomal stage of the infection. During this period, growth is also arrested. The cell cycle arrest and resume during the *B. abortus* trafficking in host cell suggest that like the model Alphaproteobacterium *Caulobacter crescentus*, these bacteria are able to block their cell cycle at the G1 phase when starvation is sensed.

The *Brucellae* are facultative intracellular pathogens, able to massively replicate in the ER, for example in chorionic trophoblasts of infected pregnant ruminants. The infection can be mimicked with cultured cells, typically HeLa cells, which are easy models to follow the intracellular trafficking of *Brucella*. The infection of HeLa cells is characterized by two successive stages. During the first stage the *Brucella* containing vacuole (BCV) is associated with endosomal-lysosomal markers such as Lamp-1 and the number of colony forming units (CFU) is stable, suggesting that bacteria do not grow, or that growth and death compensate each other. Around 10 h post-infection (PI) in HeLa cells, the bacteria are found in the ER, where they begin the proliferation until the infected cells are almost filled with bacteria.

The *Brucellae* are members of the *Alphaproteobacteria*. This taxon comprises the well-known model bacterium *C. crescentus*, studied for its differentiation and its cell cycle. An interesting feature of *C. crescentus* is the ability to block its cell cycle at the G1 phase (i.e. before the initiation of chromosomal replication) when it is in the swarmer state, a motile cell type able to explore new niches in its environment. Even if *B. abortus* and *C. crescentus* are only distantly related at the phylogenetic level within the *Alphaproteobacteria*, several key regulators of the cell cycle are conserved, as well the asymmetric position of the division site and the presence of proteins associated to bacterial new and/or old poles. We thus wondered if the cell cycle could be coordinated with the intracellular infection, and if the first stage of the intracellular trafficking was corresponding to a cell cycle arrest at a specific stage. We first showed that the frequency of dividing cells (i.e. cells with a visible constriction) was low during the first 6 h PI in HeLa cells, suggesting that cell cycle was not progressing. We confirmed this by showing that unipolar growth was strongly impaired at the same stage of the infection.

In order to differentiate the G1 versus S+G2 cell types generated during the cell cycle we developed tools to monitor the initiation of replication at the single cell level. This task was complicated by the fact that *B. abortus* bears two chromosomes, a large chromosome (chrI, 2.1 Mb) and a small chromosome (chrII, 1.2 Mb) resembling megaplasmids, with a RepABC replication/segregation system often found in *Alphaproteobacteria*. We thus constructed reporter systems to monitor the number of replication origin(s) and terminators of both chromosomes: *oriI *and *terI* for chrI, and *oriII *and *terII* for chrII. We inserted small sequences bound by proteins fused to a fluorescent tag (either YFP or CFP) close to the *ori* or *ter* sites or by localizing proteins of the segregation apparatus of *oriI* and *oriII*, ParB and RepB respectively. Observation of numerous bacteria producing both fusions showed that the two origins of replication reside in different positions within the bacterial cells (*oriII* being less anchored to the poles compared to *oriI*), and also that replication of chrI is initiated before chrII. The localization of predicted replication terminator regions confirmed the differential position and replication timing of the two chromosomes, chrII being more internal and terminating its replication and segregation sooner than chrI.

During infection, from 15 min to 6 h PI, the majority (about 75-80%) of the bacteria were in the G1 phase, contrasting with the low frequency (about 20-25%) of G1 bacteria observed in culture. Thus the endosomal stage (Lamp-1 positive) of the cellular infection correlates with a specific cell cycle arrest at the G1 phase. Furthermore, the maturation of the BCV from the endosomal markers to the ER markers correlates with the initiation of bacterial proliferation and requires the type IV secretion system VirB. Indeed, the *B. abortus virB* mutant remains in endosomal compartments and does not proliferate intracellularly. We thus compared the growth of a wild type strain and a *virB* mutant inside host cells, by characterizing their respective BCV. Surprisingly, we observed that growth and replication (but not division) of the wild type strain are resumed when bacteria are still in endosomal compartments, just before the maturation of the endosomal BCV into a proliferative BCV. The *virB* mutant was able to resume growth, but the cell cycle was almost never completed, since daughter cells were not observed, confirming that growth could be resumed in Lamp-1 positive compartments. In addition, this observation shows that the VirB system is not required to restart growth inside host cells.

Unexpectedly, the pattern of G1 enrichment at early times PI was also observed in RAW264.7 macrophages, although with a different kinetics of growth, which was resumed at 6 h PI instead of 8 h PI. We expected that macrophages would uptake actively any bacterial cell type, and these data suggest that, at least in these conditions, the G1 bacteria are able to control their internalization. Actually, it will be also interesting to test other host cells infections, with trophoblasts or activated macrophages, to detect a possible G1 arrest during the intracellular trafficking. In this context, the investigation of the cell cycle progression of other intracellular pathogens displaying a biphasic infection like *B. abortus* would be very instructive.

Altogether, these data show that the cell cycle of the *B. abortus* pathogen is coordinated with its cellular infection process. The mechanisms controlling cell cycle progression inside host cells remains to be discovered. They could for example involve the sensing of starvation, acidic pH, oxidative stress, cationic peptides, limited access to iron or "bacterial density sensing", through the release and capture of pheromones. Finally, it is tempting to establish a correlation between The G1 arrest of *B. abortus* and the G1 arrest reported for *C. crescentus* (Figure 1). Indeed, both bacteria are blocked in G1 when they face starvation conditions, and resume cell cycle when they encounter a rich environment. The transition between the environments is permitted by the high mobility of the swarmer cells in *C. crescentus*, while the VirB system of *B. abortus* allows the bacterium to reach the ER, its favourite intracellular replication niche. It is thus likely that control of cell cycle progression is adapted to fluctuating environments, in which starvation periods alternate with better conditions allowing proliferation.

**Figure 1:**
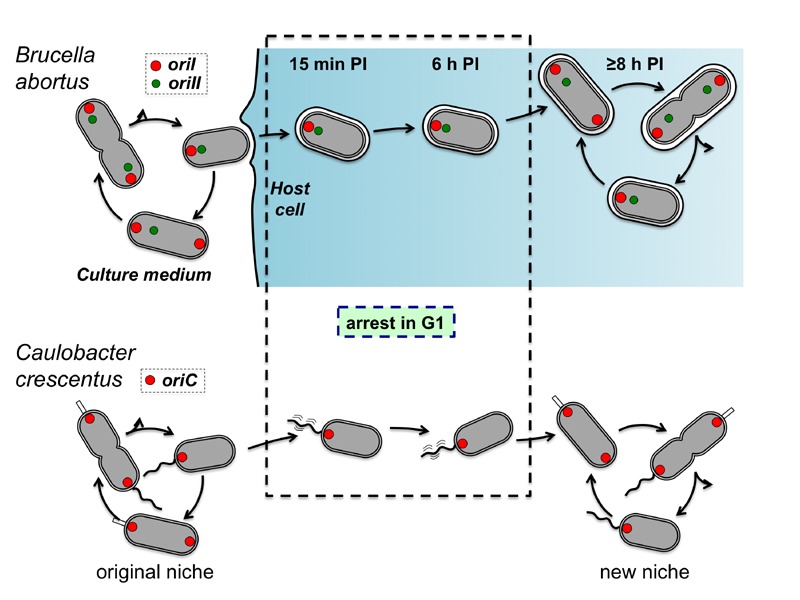
FIGURE 1: Comparison of *B. abortus* and *C. crescentus* cell cycle controls according to their lifestyle. The *B. abortus* replication origins of chromosome I (*oriI*) and chromosome II (*oriII*) are shown in red and green, respectively. The replication origin of the unique chromosome of *C. crescentus* (*oriC*) is shown in red. *B. abortus* duplicates its *oriI* before replication an segregation of its *oriII*. Inside host cells, at 15 min until 6 h post-infection (PI), the bacteria are mainly in G1 since unique spots of *oriI* and *oriII* are observed. This G1 arrest is similar to the G1 arrest observed in swarmer cells of *C. crescentus*. Thanks to their unique polar flagellum, the swarmer cells are able to move from an original niche to a new niche, in which nutrients are more abundant. In the original niche, the stalked cells are immobilized because they stick to a substrate, thanks to the holdfast at the end of their stalk (small white rectangle). In the case of *B. abortus*, the ER could be considered as a new niche in which nutrients are abundant, since this pathogen massively replicates in this organelle.

